# Evaluation of the efficacy of regorafenib treatment by location and *RAS* mutation in metastatic colon cancer

**DOI:** 10.1097/MD.0000000000042897

**Published:** 2025-06-27

**Authors:** Serdar Ata, Ahmet Gülmez, Tolga Köşeci, Özge Özalp, Timuçin Çil, Berna Bozkurt Duman

**Affiliations:** a Department of Medical Oncology, University of Health Sciences, Adana City Training and Research Hospital, Adana, Turkey; b Department of Medical Oncology, Faculty of Medicine, Çukurova University, Adana, Turkey; c Department of Medical Genetics, University of Health Sciences, Adana City Training and Research Hospital, Adana, Turkey.

**Keywords:** colon cancer, OS, PFS, regorafenib

## Abstract

The preferred approach for metastatic colon cancer is conventional chemotherapy and biological agents. Despite these treatments, most patients progress to the metastatic stage. Regorafenib inhibits various angiogenic receptor tyrosine kinases and intracellular signaling kinases. This study aimed to determine whether tumor location affects the response to regorafenib treatment in patients with colon cancer. This was a retrospective study, patients who were followed up and treated with a diagnosis of colon cancer in Adana City Training and Research Hospital Medical Oncology Clinic between January 1, 2017 and January 1, 2022 were reviewed. This study included 74 patients with adenocarcinoma, of whom 47 (63.5%) were male and 27 (36.5%) were female. Of these patients, 31 (41.9%) had left colon cancer, 10 (13.5%) had right colon cancer, and 33 (44.6%) had rectal cancer. Genetic analysis of 63 patients was available. *KRAS* wild-type and mutant tumors were detected in 31 (49.2%) and 32 (51.8%) patients, respectively. While *NRAS* wild-type was detected in all patients, no *BRAF* mutation was detected. According to *KRAS* mutation analysis, progression-free survival and overall survival of patients with *KRAS* wild-type and *KRAS*-mutant were 4.12 ± 0.4 and 3.84 ± 0.4 months (*P* = .711) and 22.1 ± 3.7 and 9.8 ± 1.5 months (*P* = .030), respectively. Unlike other studies in the literature, we found that the efficacy of regorafenib treatment in the right and left colon was not different. However, we found that this difference in efficacy was associated with the *RAS* mutation status rather than right–left colon localization. Treatment efficacy decreased in patients with *RAS*-mutant tumors. We believe that, rather than the right or left colon location, *RAS* wild-type or *RAS*-mutant status should be considered when selecting regorafenib treatment.

## 1. Introduction

According to the World Health Organization^[1]^ cancer data, colon cancer is the second most common cancer in men and women in recent years. Approximately 25% of patients have metastatic disease at initial diagnosis, while 50% to 60% of them develop metastasis during follow-up.^[2,3]^ The most preferred approach to metastatic colorectal cancer is chemotherapy including fluoropyrimidine, oxaliplatin, and irinotecan, in combination with an epidermal growth factor receptor (EGFR) inhibitor (cetuximab–panitumumab) for patients without *KRAS* mutations, and with a vascular endothelial growth factor (VEGF) inhibitor (bevacizumab, aflibercept) for patients with *KRAS* mutation^[2]^ (National Comprehensive Cancer Network Clinical Practice Guidelines in Oncology [NCCN guidelines]: colon cancer). Despite these treatments, the majority of the patients’ diseases progress.

Regorafenib is an agent that inhibits various angiogenic receptor tyrosine kinases (RTK) (vascular EGFR [“VEGFR”]-1, VEGFR-2, VEGFR-3), oncogenic RTK (c-kit, RET), stromal RTK (platelet-derived growth factor receptor-beta, fibroblast growth factor receptor-1), and intracellular signaling kinases.^[4]^ The phase 3 CORRECT study demonstrated its efficacy after first-line therapy for metastatic colon cancer. It was approved by the FDA in October 2012 and has since been used for the treatment of metastatic colon cancer.

This study aimed to determine whether tumor location affects the response to regorafenib treatment in patients with colon cancer.

## 2. Methods

This was a retrospective study, and patients who were followed up and treated with a diagnosis of colon cancer in the Adana City Training and Research Hospital Medical Oncology Clinic between January 1, 2017, and January 1, 2022, were reviewed. Ethical approval for the study was obtained from the Ethics Committee of Adana City Training and Research Hospital. Patients for whom a diagnosis of colon cancer was entered into the hospital information system were evaluated. The treatments received by patients who underwent radiological biopsy from the potential site of the primary tumor or metastasis and who were pathologically diagnosed with colon adenocarcinoma were evaluated. This study included 74 patients who received regorafenib treatment.

Right-sided colon cancers were considered to originate from the cecum, ascending colon, hepatic flexure, and proximal two-thirds of the transverse colon, whereas left-sided colon cancers were considered to arise from the distal one-third of the transverse colon, splenic flexure, descending colon, and sigmoid.^[5]^ While performing statistical analysis, the data of patients with rectal cancer were evaluated by including left-sided colon cancers.

While evaluating metastases, in which patients received regorafenib treatment, adjuvant treatments were not taken into account. All the patients were treated with irinotecan or oxaliplatin.

Genomic DNA was isolated from paraffin block sections of the patients. Mutations in *KRAS* codons 12, 13, 59, 61, 117, and 146 were evaluated using a commercial Easy *KRAS* (Diatech Pharmacogenetics) kit, and the presence of known mutations in *NRAS* codons 12, 13, 59, 61, 117, and 146 was investigated in the same DNA samples using an Easy *NRAS* (Diatech Pharmacogenetics) commercial kit. Mutations in the V600 position of the *BRAF* gene were evaluated in the same patient samples using an Easy *BRAF* commercial kit (Diatech Pharmacogenetics). Although the kit evaluates mutations in the V600 position of the *BRAF* gene, it cannot differentiate between mutations and can only detect the presence of any of these mutations. *KRAS*, *NRAS*, and *BRAF* mutation analysis results were evaluated by examining genetic laboratory records.

The data of patients eligible for the study were statistically analyzed. Treatment response to and progression of patients were determined by assessing conventional imaging reports in the hospital information system according to the Response Evaluation Criteria in Solid Tumors. The status of the patients followed up with positron emission tomography/computed tomography was determined by assessing the results.

## 3. Statistics

SPSS (Statistical Package for the Social Sciences) version 25.0 software package was used for statistical analysis of the data. Categorical measurements were summarized as numbers and percentages, and continuous measurements were presented as means and standard deviations (median and minimum–maximum where appropriate). The Shapiro–Wilk test was used to determine whether the parameters included in the study followed a normal distribution. ROC curve analysis was also performed to determine the power of the neutrophil-to-lymphocyte ratio value to predict mortality and progression-free survival. The Kaplan–Meier test and log-rank test were used for survival analysis. The level of statistical significance was set at 0.05 in all tests.

## 4. Results

The study included 74 patients, of whom 47 (63.5%) were male and 27 (36.5%) were female, with a median age of 62.5 (range, 28–90) years. Of these patients, 31 (41.9%) had left colon cancer, 10 (13.5%) had right colon cancer, and 33 (44.6%) had rectal cancer. Genetic analysis was performed in 63 patients. *KRAS* wild-type was detected in 31 (49.2%) patients and a mutation was detected in 32 (51.8%) patients. *NRAS* wild-type was detected in all patients, and there were no *BRAF* mutations. *KRAS* wild-type was detected in 26 (46.4%) patients with left-sided colon cancer and in 5 (71.4%) patients with right-sided colon cancer. Patients for whom biological therapy information was available before regorafenib treatment were evaluated. Twenty-six (47.2%) patients with left-sided colon cancer and 3 (33%) patients with right-sided colon cancer used anti-EGFR, and all patients used anti-VEGF. Immunohistochemical microsatellite instability (MSI) parameters were evaluated in patients with right- and left-sided colon cancers. While MSI-low was detected in one (2.8%) of the patients with left-sided colon cancer, all other patients were MSI-stable. MSI-stable tumors were found in all the patients with right-sided colon cancer. The neutrophil-to-lymphocyte ratio was analyzed, which was found to be 5.9 ± 11.6 in patients with left-sided colon cancer and 4.15 ± 2.5 in patients with right-sided colon cancer (*P* = .657). The evaluation of other parameters revealed no statistical differences between the 2 groups. Demographic information of the patients is presented in Table 1.

**Table 1:** Clinical and genetic characteristics of patients.

			Left colon (n = 64)	Right colon (n = 10)	*P*-value
Age	Years mean ± standart deviation		62.4 ± 13.4	59.0 ± 9.0	.331
Gender					
Male		n (%)	41 (64.1)	6 (60)	.804
Female			23 (35.9)	4 (40)	
Pre-regorafenib biological therapy		n (%)			
Anti-EGFR			9 (16.4)	–	.414
Anti-VEGF			29 (52.7)	6 (66.7)	
Anti-EGFR and Anti-VEGF			17 (30.9)	3 (33.3)	
Line of regorafenib use		n (%)			
Metastatic second-line			11 (17.7)	1 (10)	.014*
Metastatic third-line			42 (67.7)	4 (40)	
Metastatic fourth-line			9 (14.6)	4 (40)	
Metastatic fifth-line			–	1 (10)	
Metastasis location		n (%)			
Liver			16 (25)	3 (30)	.882
Liver and lung			31 (48.4)	4 (40)	
More than 2 visceral organs			17 (26.6)	3 (30)	
*KRAS*		n (%)			
Wild			26 (46.4)	5 (71.4)	.212
Mutant			30 (53.6)	2 (28.6)	
*NRAS*		n (%)	56 (100)	7 (100)	–
*BRAF*		n (%)	56 (100)	7 (100)	–
MSI		n (%)			
Low			1 (2.8)	–	.706
Stable			35 (97.2)	5 (100)	
Neutrophil-to-lymphocyte ratio		n (%)	5.90 ± 11.6	4.15 ± 2.5	.657

Anti-EGFR = epidermal growth factor receptor, Anti-VEGF = anti-vascular endothelial growth factor, MSI = microsatellite instability.

* *P* < .05.

**Figure 1.**
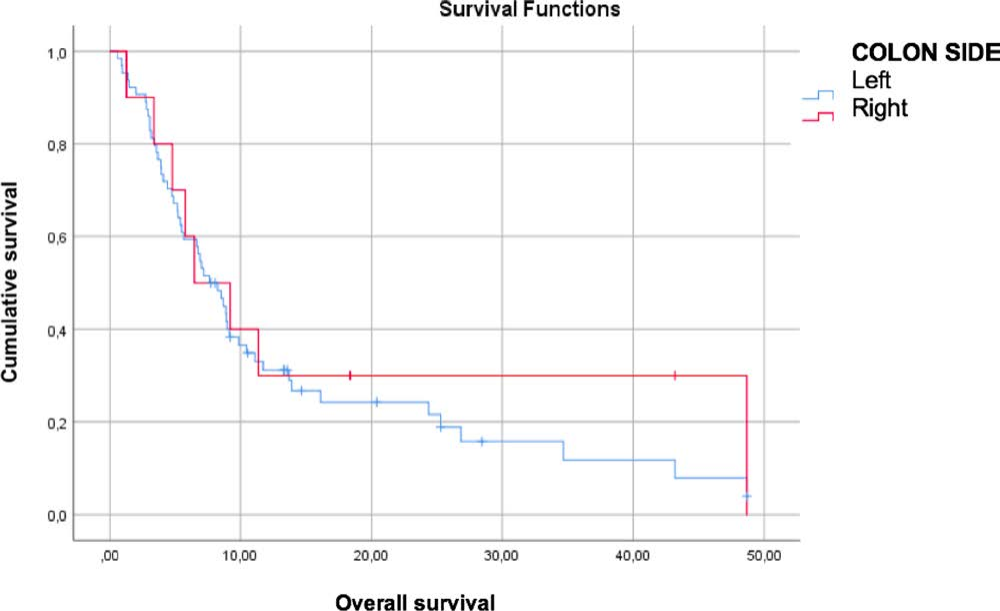
Kaplan–Meier survival curves (months) according to tumor location.

**Figure 2.**
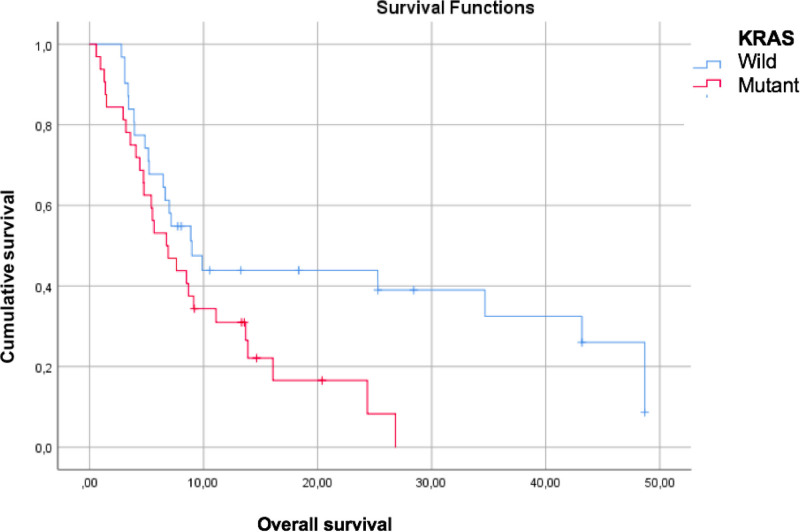
Kaplan–Meier survival curves (months) according to *KRAS* mutation.

The mean overall survival (OS) and progression-free survival (PFS) of all patients were 14.8 ± 3.9 and 3.9 ± 0.3 months, respectively. The PFS of patients with left- and right-sided colon cancer was 3.76 ± 0.3 and 4.54 ± 0.8 months (*P* = .235) respectively (Fig. 1). The OS of patients with left- and right-sided colon cancer was 13.9 ± 1.8 and 18.8 ± 6.7 months (*P* = .516), respectively (Fig. 1). According to *KRAS* mutation analysis, PFS of patients with *KRAS* wild-type and *KRAS*-mutant were 4.12 ± 0.4 and 3.84 ± 0.4 months (*P* = .711) respectively (Fig. 2). The OS of patients with *KRAS* wild-type and *KRAS*-mutant was 22.1 ± 3.7 and 9.8 ± 1.5 months (*P* = .030), respectively (Fig. 2). The OS and PFS analyses of the patients demonstrated longer OS and PFS in patients with low neutrophil/lymphocyte ratios (*P* = .027; *P* = .021), which was statistically significant. In contrast, location, biological treatment, metastatic line of regorafenib use, number of visceral organ metastases, location of metastases, and sex were not significantly correlated with PFS and OS (*P* > .05) (Table 2).

**Table 2:** Progression-free survival and overall survival of patients.

	PFS Mean ± SD	95% CI		*P*-value	OS Mean ± SD	95% CI		*P*-value
		Lowest	Highest			Lowest	Highest	
Colon cancer location								
Left	3.76 ± 0.3	3.25	4.26	.235	13.9 ± 1.8	9.9	17.9	.516
Right	4.54 ± 0.8	3.05	6.04		18.8 ± 6.7	5.8	31.9	
Pre-regorafenib biological therapy								
Anti-EGFR	3.59 ± 0.8	2.10	5.09	.730	28.1 ± 7.7	13.1	43.1	.061
Anti-VEGF	4.04 ± 0.4	3.23	4.85		10.1 ± 1.8	6.6	13.6	
Anti-EGFR and Anti-VEGF	3.79 ± 0.5	2.90	4.67		20.1 ± 4.7	10.9	29.4	
Metastasis location								
Liver	4.22 ± 0.5	3.18	5.27	.813	17.1 ± 4.2	8.9	25.2	.788
Liver and lung	3.87 ± 0.3	3.21	4.53		13.7 ± 2.6	8.5	18.8	
More than 2 visceral organs	3.59 ± 0.5	2.56	4.61		15.3 ± 3.9	7.5	23.1	
*KRAS*								
Wild	4.12 ± 0.4	3.33	4.90	.711	22.1 ± 3.7	14.8	29.4	.030*
Mutant	3.84 ± 0.4	3.03	4.65		9.8 ± 1.5	6.8	12.8	
Gender								
Male	3.78 ± 0.3	3.11	4.45	.602	13.6 ± 2.3	9.2	18.1	.424
Female	4.10 ± 0.4	3.40	4.79		16.8 ± 3.6	9.8	23.8	
Age								
75 yr and under	3.80 ± 0.3	3.26	4.34	.623	15.8 ± 2.3	11.2	20.3	.751
Over 75 yr	4.39 ± 0.6	3.19	5.58		10.5 ± 2.1	6.4	14.6	
NLR								
Low	4.17 ± 0.3	3.62	4.72	.021*	15.3 ± 2.1	11.1	19.5	.027
High	2.75 ± 0.4	1.92	3.57		10.9 ± 4.3	2.4	19.4	

Anti-EGFR = epidermal growth factor receptor, Anti-VEGF = anti-vascular endothelial growth factor, NLR = neutrophil-to-lymphocyte ratio, OS = overall survival, PFS = progression-free survival, SD = standard deviation.

* *P* < .05.

## 5. Discussion

Our study demonstrated that the efficacy of regorafenib treatment was not different between right- and left-sided colon cancers. However, we revealed that the efficacy differed according to *RAS*-mutant status. Treatment efficacy decreased in patients with *RAS*-mutant tumors.

The introduction of new drugs for metastatic colon cancer has prolonged patient survival. Although surgery is the best treatment method for patients with early stage disease, conventional chemotherapy and targeted therapies are the treatment of choice for patients with metastatic colon cancer. Treatment with regorafenib, a multi-tyrosine kinase inhibitor, has come to the fore in patients who progress to these treatments. Studies on the use of regorafenib therapy in metastatic colon cancer have reported PFS ranging from 1.9 to 3.2 months and OS ranging from 6.4 to 9.8 months.^[4,6–8]^

Evaluation of the VEGF pathway according to colon location has shown that it is more active in the left colon than in the right colon.^[9,10]^ Although it has been suggested that regorafenib, which primarily shows its effect via the VEGF receptor, may have different efficacies in right- and left-sided colon cancers, our study demonstrated no effect of colon cancer location on treatment response. This indicates that multiple factors affect the response to regorafenib. Both PFS and OS were longer in patients with *KRAS* wild-type tumors than in those with *KRAS*-mutant tumors. The prolongation of OS was also statistically significant.

The question of which patients will benefit more from regorafenib treatment, which has less efficacy than the previous lines, and patient selection becomes important.^[11–14]^ Previous studies have shown that patient age, previous targeted treatments, location of metastasis, time to metastasis after adjuvant treatment, drug dosing schedule, and location affect the treatment response. Our study revealed that *RAS* mutation status rather than colon cancer location may predict response to regorafenib treatment.

Considering all patients, the mean PFS was 3.9 ± 0.3 months and the mean OS was 14.8 ± 3.9 months in our study. The CORRECT study by Grothey et al^[4]^ reported PFS of 1.9 months and an OS of 6.4 months. Although regorafenib was initiated at a dose of 160 mg in the CORRECT study, we initiated it at a low dose in our clinic and increased the dose according to the toxicity profile. As it takes time to reach the effective dose, we performed an interim evaluation for our patients at week 12. As the treatment was started at an effective dose in the CORRECT study, interim evaluation was performed at week 8. Therefore, we believe that the difference in PFS was related to interim evaluation time. We attribute the difference in OS to the fact that the majority of the patients in our study received regorafenib treatment in the third line, while in the CORRECT study, they received the treatment in the fourth line or further.

A study by S.E. Yoon et al^[15]^ found a PFS of 1.9 months and 2.6 months for right- and left-sided colon cancers, respectively (*P* = .04). The evaluation of subgroups revealed that this difference was not present in patients with *KRAS* mutations, whereas it was statistically significant in patients without *KRAS* mutations (*P* = .04). In our study, PFS was 4.54 ± 0.8 months and 3.76 ± 0.3 months for right- and left-sided colon cancers, respectively (*P* = .235). Under normal circumstances, the rate of *KRAS* wild-type is expected to be higher in left-sided colon cancer than in right-sided colon cancers.^[16]^ Yoon et al^[15]^ found a statistically higher rate of *RAS* wild-type in patients with left-sided colon cancer than in those with right-sided colon cancer, with a longer PFS value in left-sided colon cancers (*P* = .04). In our study, both the rate of *RAS* mutation and PFS were similar in left- and right-sided colon cancers. This suggests that the response to regorafenib treatment may be related to *RAS* mutation status rather than the intensity of the VEGF pathway.

A study by E.V. Cutsem et al^[4]^ found a PFS of 2.5 months and 2.8 months in patients with and without *KRAS* mutations, respectively, with no statistically significant difference. In our study, PFS was 3.84 ± 0.4 months in patients with *KRAS* mutation and 4.12 ± 0.4 months in those without *KRAS* mutation (*P* = .711). As mentioned above, the *RAS* mutation status may affect the response to regorafenib treatment.

A study by Saab et al^[8]^ aimed to increase drug tolerance and reduce side effects by starting regorafenib treatment at a dose of 80 mg and increasing it by 40 mg weekly. This study showed that the percentage of patients who received 3 cycles of treatment was 43.26% compared to the group that received 160 mg regorafenib (*P* = .043). The comparison of the dose-titrated group with the patients who were initiated on 160 mg revealed an PFS of 2.8 months and 2 months (*P* = .38) and OS of 9.8 months and 6 months (*P* = .12), respectively. We initiated regorafenib treatment at a dose of 80 mg and increased the dose to 160 mg in weekly increments. The patients in our study had longer PFS and OS. This can be explained by the interim evaluation of the patients in our study performed at month 3, while in another study, it was performed at month 2. With earlier evaluations, the progression may be detected earlier. Patients with 3 or more metastatic sites in our study accounted for 27% of the patients included in the study, whereas this rate was 67% in T.S.B. Saab et al This may explain the longer PFS and OS.

In a study by Petrioli et al^[17]^ in patients over 75 years of age, 160 mg/d regorafenib was administered for 2 weeks to increase drug tolerance. The drug was administered for 3-weeks cycles with a 1-week break, with the aim of decreasing toxicity and increasing drug tolerance in elderly patients. This study reported an OS of 8.9 months. In addition, the OS values were found to be longer with the 3-weeks use of 160 mg/d regorafenib and 1-week break than with the 4-weeks cycle (*P* < .001). In our study, OS was 8.12 ± 1.2 months in patients over 75 years of age, which was shorter than that in the general population. In this age group, choosing the method of 2-weeks treatment and 1-week break, which reduces drug toxicity and increases the continuity of drug use, and accordingly increases drug efficacy, may be a more effective method in this age group.

In conclusion, unlike other studies in the literature, our study demonstrated that the efficacy of regorafenib treatment did not differ between right- and left-sided colon cancers. However, our results revealed that this efficacy difference was associated with *RAS* mutation status rather than the right or left colon location. Treatment efficacy decreased in patients with *RAS*-mutant tumors. We believe that, rather than the right or left colon location, *RAS* wild-type or *RAS*-mutant status should be considered when selecting regorafenib treatment.

## 6. Limitations

The ECOG performance scores of the patients could not be evaluated because the patients’ data were accessed from the hospital operating system. The limited number of patients with right colon cancer and the absence of patients with *NRAS–BRAF* mutations are limitations of our study.

## Author contributions

**Conceptualization:** Serdar Ata, Özge Özalp, Timuçin Çil, Berna Bozkurt Duman.

**Data curation:** Serdar Ata, Tolga Köşeci.

**Formal analysis:** Serdar Ata, Ahmet Gülmez.

**Investigation:** Ahmet Gülmez, Tolga Köşeci, Özge Özalp.

**Methodology:** Tolga Köşeci.

**Resources:** Ahmet Gülmez, Özge Özalp.

**Software:** Serdar Ata, Tolga Köşeci.

**Validation:** Serdar Ata, Tolga Köşeci.

**Visualization:** Serdar Ata, Ahmet Gülmez.

**Writing – original draft:** Serdar Ata, Tolga Köşeci.

**Writing – review & editing:** Serdar Ata, Özge Özalp, Timuçin Çil, Berna Bozkurt Duman.
